# A73 EFFECT OF FECAL IMMUNOCHEMICAL TEST CUT-OFF LEVELS ON ADENOMA DETECTION RATE: A SYSTEMATIC REVIEW AND META-ANALYSIS

**DOI:** 10.1093/jcag/gwac036.073

**Published:** 2023-03-07

**Authors:** M Zarandi-Nowroozi, M Taghiakbari, A Barkun, H Pohl, B Nauche, M Chagnon, D von Renteln

**Affiliations:** 1 University of Montreal Hospital Center (CHUM); 2 University of Montreal Hospital Research Center (CRCHUM); 3 McGill University Health Centre (MUHC), Montreal, Canada; 4 Dartmouth Geisel School of Medicine, Hanover, United States; 5 University of Montreal, Montreal, Canada

## Abstract

**Background:**

Adenoma detection rate (ADR) is higher after a positive fecal immunochemical test (FIT) compared to direct screening colonoscopy.

**Purpose:**

This meta-analysis evaluated how ADR, the rates of advanced adenoma detection (AADR), colorectal cancer detection (CDR), and sessile serrated lesion detection (SSLDR) are affected by different FIT positivity thresholds.

**Method:**

We searched MEDLINE, EMBASE, CINAHL, and EBM Reviews databases for studies reporting ADR, AADR, CDR, and SSLDR according to different FIT cut-off values in asymptomatic average-risk individuals aged 50 to 74 years old. Data were stratified according to sex, age, time to colonoscopy, publication year, continent, and FIT kit type. Study quality, heterogeneity, and publication bias were assessed.

**Result(s):**

Overall, 4280 articles were retrieved and fifty-seven studies were included (332,281 FIT-positive colonoscopies; mean cecal intubation 96.2%; mean age 60.7 years; male 52.1%). Mean ADR was 55.9% (95% CI 53.2% – 58.6%), while mean AADR, CDR, and SSLDR were 27.2% (95% CI 24.3% – 30.1%), 5.4% (95% CI 4.7% – 6.1%), and 3.0% (95% CI 1.7% – 4.6%), respectively. For each 20 μg Hb/g increase in FIT cut-off level, ADR increased by 2.87% (95% CI 1.70% – 4.05%, p < 0.01), AADR increased by 3.90% (95% CI 2.76% – 5.05%, p < 0.01) and CDR by 1.46% (95% CI 0.66% – 2.24%, p < 0.01). Many detection rates were greater amongst males and Europeans.

**Conclusion(s):**

ADRs in FIT-positive colonoscopies are influenced by the adopted FIT positivity threshold, and identified targets, importantly, proved to be higher than most current societal recommendations.

**Please acknowledge all funding agencies by checking the applicable boxes below:**

None

**Disclosure of Interest:**

None Declared

